# A national study of in-hospital preparedness for Mass Casualty Incidents and disasters

**DOI:** 10.1007/s00068-024-02685-7

**Published:** 2025-01-15

**Authors:** Kristina Stølen Ugelvik, Øyvind Thomassen, Geir Sverre Braut, Thomas Geisner, Janecke Engeberg Sjøvold, Carl Montán

**Affiliations:** 1 Regional Trauma Centre, Haukeland University Hospital, Bergen, Norway; 2 University of Bergen, Bergen, Norway; 3 Helicopter Emergency Medical Service, Haukeland University Hospital, Bergen, Norway; 4 Norwegian Air Ambulance Foundation, Oslo, Norway; 5 Stavanger University Hospital, Stavanger, Norway; 6 Western Norway University of Applied Sciences, Bergen, Norway; 7 Gastrointestinal Surgery Department, Haukeland University, Bergen, Norway; 8 Department of Molecular Medicine and Surgery, Karolinska Institutet, Stockholm, Sweden

**Keywords:** Mass casualty incidents, Hospital preparedness, Hospital surge capacity, Disaster preapredness

## Abstract

**Purpose:**

The current geopolitical situation and climate changes accentuate the importance of health preparedness. The aim was to examine the in-hospital preparedness for Mass Casualty Incidents (MCI) and Major Incidents (MI) on a national level.

**Method:**

A web-based, cross-sectional study of in-hospital preparedness for MCI/MI in Norway. All hospitals with trauma function were included with 3 defined representatives, excluding hospitals without trauma function. The survey consisted of 63 questions covering: MCI/MI organisation, education, plans, Surge Capacity, triage and supply management.

**Results:**

The study had a response rate of 97/112 (87%), representing 35/38 (92%) of the included hospitals. Contingency responsible respondents (CRR) reported that 27/34 (80%) of the hospitals had a contingency responsible function/role and 29/34 (85%) had a Disaster Preparedness Committee. Among CRR, formal MCI/MI education 5/34 (15%) and MCI/MI training 9/34 (26%) was completed. Further, 87/97 (90%) had an all-hazard contingency plan. MCI/MI exercise within the last 2 years was reported by 63/97 (65%). Surge Capacity was assessed within the last 5 years at 6/35 (17%) of the hospitals. MCI/MI material storage was reported by 56/97 (58%).

**Conclusion:**

Many key aspects of contingency work were found to be well-established. MCI/MI education and training for roles/functions was missing in most hospitals. Areas of improvement detected included Surge Capacity and emergency storage. The results suggest a need for national minimum standards and requirements. National in-hospital MCI/MI preparedness could be monitored by a web-based survey, providing information of pan-European relevance.

**Supplementary Information:**

The online version contains supplementary material available at 10.1007/s00068-024-02685-7.

## Introduction

Climatic changes, terrorist attacks and the current geopolitical situation have placed Mass Casualty Incidents (MCI) and Major Incidents (MI) preparedness back on the public and political agenda. Europe is currently experiencing a war. According to NATO, “each NATO member country needs to be resilient in order to withstand a major shock such as a natural disaster, failure of critical infrastructure, or a hybrid or armed attack” [1]. There are still military clinics and hospitals in Europe, but some European countries like Norway depends on the civilian health system. NATO acknowledge the civilian preparedness as an important part of the joint defence [1]. The European Health Emergency Preparedness and Response Authority has CBRN as one of three focus areas, the others are related to pandemic and antibiotic resistance [2]. Evaluations after the Covid-19 Pandemic revealed a need for improved coordination and cross-border focus in the pan-European preparedness. Deficiencies in production and supplies for protective personal equipment (PPE) and pharmaceuticals were exposed [2]. The response plan for burn MCI within the European Union (EU) Civil Protection Mechanism and the NIGHTINGALE project focusing on first responder skills in prehospital MCI management are examples of cross-border cooperation within Europe [3, 4].

MCI/MI by nature and definition imply great challenges to in-hospital health care systems [5]. Organisation, planning, education, training, exercises and evaluation routines can impact the outcome of MCI/MI [6–13]. Thorough knowledge about the Surge Capacity, capacity limitation factors and means to increase Surge Capacity is a crucial part of the in-hospital contingency work [14, 15]. Hick et al. identified the 4S`s that impact hospital Surge Capacity; system, space, staff, and supplies [14]. Deficiencies in the preparedness system and/or plans, lack of physical space to receive large inflow of patients, lack of competent staff to manage the patients and critical supplies such as ventilators and blood supply can negatively affect patient outcome. Different tools and methods can be utilised to evaluate and monitor in-hospital MCI/MI preparedness on a local, regional and national level [16–22].

Norway is sparsely populated with a long coastline, varying distances, road conditions and weather conditions that can affect sea, road and air ambulance traffic from the scene of an incident to the hospital. Hospital care is provided through 4 Regional Health Authorities (RHA). Each of the RHA has a Regional Trauma Centre (TC) as well as several Acute Hospitals with trauma function/Non-trauma Centre (NTC) to manage trauma patients [23]. A national trauma plan (NTP) defines quality of care indicators, establishes minimum competency requirements and aims to reduce differences between the hospitals and health regions [23]. The NTP contains criteria regarding which trauma patient to be taken to TC and NTC during normal situations as well as during MCI/MI and criteria’s for member of the trauma team [23]. There is considerable variation in trauma burden among Norwegian hospitals [23]. No national in-hospital triage guideline exists as the size of the hospitals and the resources available varies.

Currently there is no national method for systematically following up the in-hospital MCI/MI preparedness work in Norway. The aim of this study was to describe the current preparedness for MCI/MI among Norwegian Trauma Hospitals using an adapted national standardised survey based on the methodology applied by Söderin et al. in a Swedish study of MCI/MI preparedness on a national level [17].

## Methods

**Fig. 1:**
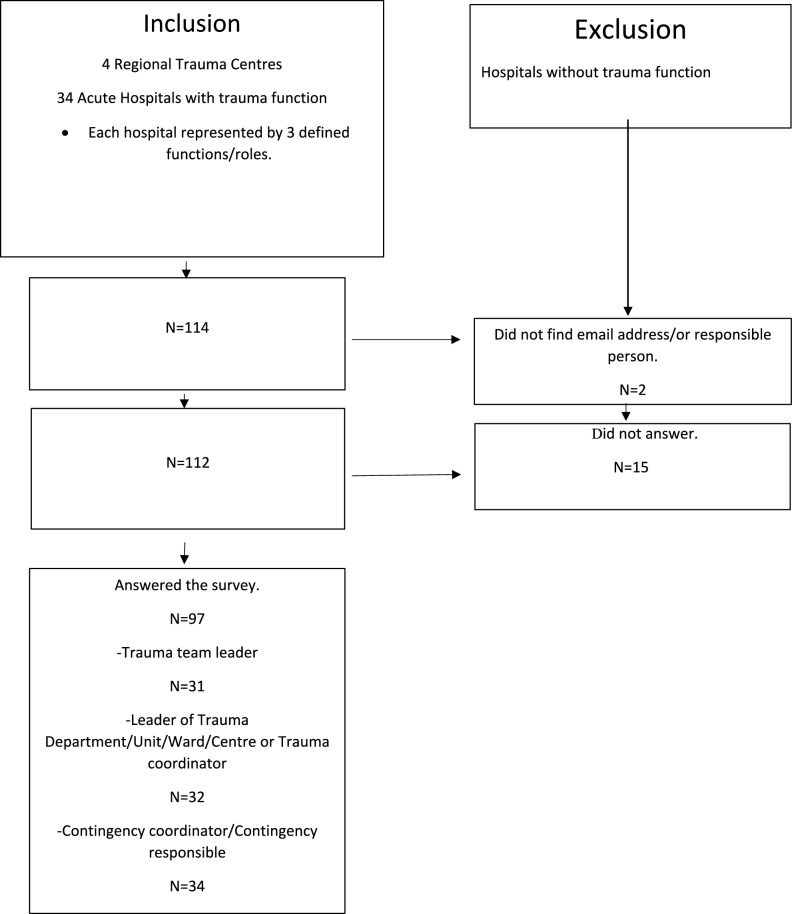
Flowchart

The study was designed as a descriptive cross-sectional study of the Norwegian in-hospital preparedness for MCI/MI. All 4 Regional TCs and 34 NTCs in Norway were included in the study. Hospitals without trauma function were excluded. Each included hospital was asked to nominate 3 participants for the study to get a representative view of the preparedness work (Fig. 1):

- Trauma team leader
- Leader trauma unit/ward/centre or Trauma coordinator
- Contingency coordinator/Contingency responsible responder (CRR)

A web-based survey was used to gain information regarding the following focus areas in MCI/MI in-hospital preparedness: organisation, education, training, contingency plan, exercises, triage, Surge Capacity and supply management. The survey consisted of 63 questions and was adapted to Norwegian context based on the methodology used in the Swedish study conducted by Soderin et al. [17].

As some questions were deemed relevant or requiring knowledge based on the function of the respondents, some answers were reported by all respondents and others by one function only.

Information about the study was sent by email 01.09.2023 to all Norwegian NTCs and TCs through trauma coordinators. Trauma coordinators at all TCs and NTCs were asked to identify 3 respondents representing their hospital covering the predefined functions/roles. Three answers per hospital was requested. The respondents were encouraged to discuss with colleagues and search for information. For those who had several roles in their organisation it was specified in the cover letter which function to represent. Data was collected in the period 23.10.2023 to 27.11.2023. Those that did not answer were reminded by phone calls and emails. Some participants were missed due to lack of names or contact information, and some did not answer despite follow up (Fig. 1).

Analysis of quantitative data was performed by exporting data to SPSS Statistics for Windows, Version 26.0 (IBM, New York, NY, US) and Microsoft Office 365 Excel Version 16.0 (Microsoft Corporation, Redmond, WA, US). The percentage distribution was calculated and visualised in tables.

## Results

A total of 97/112 (87%) representatives of the 3 predefined functions at the invited hospitals answered the survey: 31 trauma team leaders, 8 Leaders of trauma department/centre, 24 trauma coordinators and 34 CRR. The 4 TCs were represented by 12/12 respondents, and of the NTCs 31 out of 34 (91%) responded, as 85/112 (76%) answered the survey (Fig. 1). All Health Trusts (19/19) responsible for in-hospital specialist care were represented. The three hospitals that did not answer the survey, all belonged to the same RHA 

### Organisation and MCI/MI competency

According to 27/34 (79%) of the CRR, the hospital had a contingency coordinator or function/role responsible for disaster preparedness, and 18/34 (53%) had dedicated time to perform the duty. The contingency function was most commonly occupied by a registered nurse, as stated by 15/34 (44%) of the CRR. Of the CRR, 5/34 (15%) had either formal MCI/MI education or training in MCI/MI 9/34 (26%) (Table 1). Among the CRR at TC, 1/ 4 (25%) had formal education and 2/4 (50%) relevant training or other contingency relevant background, compared to 4/30 (13%) formal MCI/MI education and 7/30 (23%) MCI/MI training or other relevant background among NTC.

**Table 1:** Organisation of contingency work, relevant MCI/MI education and training reported by Contingency responsible respondents

Organisational role	Organisation/role/education/working model	N (%) N = 34
Contingency coordinator or role/function as contingency responsible	Person with dedicated time to perform duty as contingency responsible role/function at the hospital	18/34 (53%)
	Contingency responsible role/function with formal education in disaster and emergency preparedness	5/34 (15%)
	Contingency responsible role/function with training in disaster and emergency preparedness	9/34 (27%)
Disaster Preparedness Committee	Established Disaster Preparedness Committee at the hospital	29/34 (85%)
	Reported “No formal disaster education or training requirements” for members of the Disaster preparedness Committee	21/34 (62%)
Hospital Command Group	Reported “No formal staff methodology training requirements” for members of the Hospital Command Group	19/34 (55%)
	Use defined working model during MCI/MI	14/34 (41%)

A medical doctor with disaster preparedness responsibility was reported by 3/4 (75%) of the CRR at TC and 16/30 (53%) at NTC. None of the CRR at TC reported that the disaster preparedness responsible medical doctor had any formal MCI/MI education or training, whereas 1/30 (3%) of the CRR at NTC reported that the medical doctor had formal MCI/MI education and 5/30 (17%) reported other relevant disaster training.

The majority of the CRR (29/34, 85%) reported having a Disaster Preparedness Committee at their hospital. All TC (4/4) and 25/30 (83%) of the NTC had established a Disaster Preparedness Committee. No formal MCI/MI education or training was required for the members of the Contingency Preparedness Committee as reported by 21/34 (62%) of the CRR (Table 1).

The Hospital Command Group (HCG) was reported to follow a defined working model during MCI/MI according to 14/34 (41%) of the CRR, 12/34 (35%) answered no working model used and 8/34 (24%) “did not know”. According to the CRR, HCG had no training in staff methodology as reported by 19/34 (56%) and 8/34 (24%) responded that all members of the HCG had staff methodology training (Table 1).

Fulfilment of NTP requirement competency for all trauma team leaders was reported by 11/31 (35%) of the responding trauma team leaders. Furthermore, 14/31 (45%) reported that most of the trauma team leaders fulfilled the national requirements. Among TC trauma team leaders, 2/4 (50%) reported that all trauma team leaders fulfilled NTP requirement, compared to 17/27 (63%) of NTC trauma team leaders. Variance in the fulfilment of trauma team leader requirements was detected between the RHAs (Table 2). Certification of trauma team leaders was conducted at the hospitals of 10/31 (32%) of the trauma team leaders.

**Table 2:** Regional Health Authorities (RHA), trauma team leader competency and contingency plans

	RHA 1 N = 20	RHA 2 N = 18	RHA 3 N = 21	RHA 4 N = 38
All Trauma team leader fulfilled NTP requirements	10/20 (50%)	13/18 (72%)	2/21 (10%)	15/38 (39%)
Contingency responsible role/function with established time for contingency work	9/20 (45%)	15/18 (83%)	11/21 (52%)	18/38 (47%)
Contingency plans for MCI/MI updated within the last 12 months	10/20 (50%)	15/18 (83%)	4/21 (19%)	17/38 (45%)
Activation of contingency plan for MCI/MI within the last 2 years	8/20 (40%)	7/18 (39%)	2/21 (10%)	11/38 (29%)
MCI/MI exercises yearly or more frequently	16/20 (80%)	7/18 (39%)	8/21 (38%)	23/38 (61%)
MCI/MI exercise participation for Hospital Command Group within last 12 months	16/20 (80%)	8/18 (44%)	5/21 (24%)	17/38 (45%)
Evaluation after activation of contingency plan	11/20 (55%)	8/18 (44%)	7/21 (33%)	16/38 (42%)

Presence of a trauma coordinator or role/function as trauma coordinator at the hospital, was reported by 31/32 (97%) of the respondents in the category leader of trauma department or trauma coordinator.

### Contingency plans

An overall all-hazard contingency plan for the hospital was reported by 87/97 (90%) of the respondents. The overall all-hazard contingency plan was revised within the last 12 months according to 20/34 (59%) of the CRR and 27/34 (79%) was revised within the last 2 years (Table 3). Variances among the RHAs were detected (19–83%) (Table 2). Based on the CRR answers, half of the TCs had revised the all-hazard contingency plan within the last year compared to 18/30 (60%) among NTCs. The all-hazard contingency plan contained less than 11 pages according to 31/97 (32%) of the respondents. Accordingly, the contingency plan was coordinated with nearby hospitals contingency plans as stated by 59/97 (61%). Regarding local requirements, all employees were expected to read the contingency plan as reported by 36/97 (37%), 22/97 (23%) stated that all new employees should read the contingency plan and 21/97 (22%) replied that they either “did not know” or that there were no requirements at their hospital. MCI was the most common scenario described in the contingency plan (85/97 (88%)), followed by CBRNE (80/97 (82%)), fire (73/97 (76%)) and epidemics (71/97 (73%)).

**Table 3:** Contingency plan and MCI/MI Exercises

	Acute Hospital with trauma function (NTC) N = 85	Regional trauma centre (TC) N = 12	Contingency responsible/ function/ contingency coordinator (CRR) N = 34
All hazard contingency plan updated last 12 months	39/85 (46%)	7/12 (58%)	20/34 (59%)
Activation of contingency plan for MCI/MI < 2 years	24/85 (28%)	4/12 (33%)	12/34 (35%)
MCI/MI exercises yearly or more frequently	47/85 (55%)	7/12 (58%)	21/34 (62%)
MCI/MI exercise < 1 year with Hospital Command Group participation	40/85 (47%)	6/12 (50%)	21/34 (62%)
Evaluation after activation of contingency plan	36/85 (42%)	6/12 (50%)	20/34 (59%)

Activation of the MCI/MI contingency plan within the last 2 years was reported by 28/97 (29%) of respondents, among TC respondents 4/12 (33%) and NTC respondents 24/85 (28%) (Table 3). Within the last 5 years, 49/97 (51%) had activated the MCI/MI contingency plan, for TC 6/12 (50%). Further, 5/97 (5%) of the respondents replied that the MCI/MI contingency plan had never been activated. Variances were detected among the RHAs regarding activation of contingency plan (10–40%) (Table 2).

### Exercises

Of the respondents, 54/97 (56%) stated that the hospital had conducted a disaster exercise yearly or more frequently and 63/97 (65%) within the last 2 years. Variance in the MCI/MI exercise frequency was detected among the RHAs (38–80%) (Table 2). The HCG participated in MCI/MI exercise(s) within the last year according to 46/97 (47%) of the respondents. Insignificant differences in participation of HCG in MCI/MI exercises were found comparing NTC (40/85 (47%)) and TC (6/12 (50%)) (Table 3). MCI/MI exercises involving coordination between the respondent’s hospital and nearby hospitals was reported by 42/95 (44%) of the respondents.

### Evaluation

Activation of the MCI/MI contingency plan resulted in an evaluation as stated by 42/97 (43%) of the respondents. Accordingly, 30/97 (31%) of the respondents mentioned that the evaluation process resulted in revision of the contingency plan in case of deficiencies and 12/97 (12%) answered that the plan was functioning well, that no revision was needed.

After MCI/MI exercises, 41/97 (42%) reported that a structured evaluation was conducted, 22/97 (23%) reported that some other evaluation method was used and 14/97 (14%) used a survey as evaluation tool.

### Surge capacity

Among the respondents 34/97 (35%) answered that Surge Capacity/maximum capacity estimation for MCI/MI existed at their hospital. Further, 11/97 (11%) described how the Surge Capacity estimation was conducted. Within the last 5 years, Surge Capacity assessment was performed at 6/35 (17%) of the responding hospitals. None of the TCs had performed a Surge Capacity estimation within the last 5 years (Table 5). No Surge Capacity estimation had been conducted within the last 5 years in 2 of the RHAs (Table 6). Surgical theatre and ICU capacity was the most frequent estimated Surge Capacity limiting factor (Table 7).

**Table 5:** Acute Hospital with trauma function and Regional Trauma Centre regarding Surge Capacity and disaster storage

	Acute Hospital with trauma function (NTC) N = 85	Regional trauma centre (TC) N = 12
Surge Capacity estimation within the last 5 years	8/85 (9%)	0/12 (0%)
Disaster storage	49/85 (58%)	7/12 (58%)

**Table 6:** Surge Capacity and disaster storage among Regional Health Authorities

	RHA 1 N = 20	RHA 2 N = 18	RHA 3 N = 21	RHA 4 N = 38
Surge Capacity/max capacity estimation	9/20 (45%)	8/18 (44%)	7/21 (33%)	10/38 (26%)
Surge Capacity estimation within last 5 years	3/20 (15%)	5/18 (28%)	0/21 (0%)	0/38 (0%)
Disaster storage	13/20 (65%)	12/18 (67%)	13/21 (62%)	18/38 (47%)

**Table 7:** Top 7 Included factors in the Surge Capacity estimation (multiple answer question)

	N (%)
Surgical theatre	11/12 (92%)
ICU	10/12 (83%)
ED	9/12 (75%)
Ward beds	9/12 (75%)
Intermediate care beds	9/12 (75%)
Human resources	9/12 (75%)
Radiology	7/12 (58%)

An existing plan for how and when patients from an MCI/MI should be transferred to other hospital(s) was mentioned by 61/97 (63%) of the respondents. Regarding strategies among Norwegian hospitals to deal with high influx of trauma patients with minor to moderate injuries; 42/97 (43%) placed them in the same ward, whereas 32/97 (33%) spread the patients in different wards. In addition, 63/97 (65%) reported that the hospital had a plan for how to increase the hospital`s capacity to admit patients, 24/97 (25%) “did not know” and 8/97 (8%) answered no plan and 2/97 (2%) did not answer.

### Triage

The entrance of the emergency department (46/97 (48%)) and the emergency department (36/97 (37%)) were the most frequent locations for in-hospital MCI/MI primary triage. A variety in triage methods were reported by the respondents; ATLS, SATS, RETTS, National guidance for prehospital triage.

### Supply management

A disaster storage for MCI/MI was found at the hospital of 56/97 (58%) of the respondents and 21/56 (38%) answered that the storage was upgraded on a yearly basis. The “did not know” option regarding disaster storage was chosen by 28/96 (29%). No significant differences regarding disaster storage were detected between TC and NTC (Table 5). Variances were detected among RHA 18/38 (47%)–12/18 (67%) (Table 6). The stock contents of the disaster storage were defined in the contingency plan and in accordance with the max capacity of the hospital as stated by 24/56 (43%) of the respondents with a hospital disaster storage. Accordingly, 22/56 (39%) “did not know” which criteria was used to stock up the disaster storage.

## Discussion

Although key elements in the contingency organisation, planning and exercises were in place, several areas of improvements in the Norwegian in-hospital MCI/MI preparedness were detected. The deficiencies and variances of the preparedness work calls for national minimum requirements and standards in the in-hospital contingency work and follow up routines.

Results suggests that there is room for competency improvement for central roles/functions responsible for in-hospital MCI/MI preparedness in Norway. There are no national minimum requirements for the contingency responsible role/function or members of the Disaster Preparedness Committee to benchmark towards, as opposed to trauma team leaders where the NTP defines minimal competency [23]. Our results correspond with Jørgensen et al., detecting a deficiency among Norwegian TC/NTC to meet the NTP requirements for the trauma team leaders, indicating the need for follow up routines [18]. Challenges regarding MCI preparedness competency and training are not limited to Scandinavia [24–26]. The NIGHTINGALE workgroup detected a shortage of exposure among European health workers to penetrating trauma, highlighting the need for targeted MCI education [4].

All Norwegian trauma hospitals are mandated by law to have a contingency plan [27, 28]. Although, most Norwegian trauma hospitals comply, the percentage is slightly lower than the findings in Sweden [17]. Gabbe et al. found variances ranging from 60–100% regarding all-hazard contingency plans among major TC in Canada, Australia, England and New Zealand [16]. A contingency plan should be easy to access and understand for the employees having a role in an MCI/MI situation [5]. Based on our study, many of the respondents had an all-hazard contingency plan containing more than 11 pages. Not all hospitals had requirements regarding whom should read the contingency plan. Jørgensen et al. found that 50% of the Norwegian health care workers had studied the MCI/MI plan within the last 6 months [18]. Complicated contingency plans spanning many pages, as well as lack of requirements and follow up routines of employees can be possible explanations. Experiences gained from the Medical Evacuation response of Ukraine patients through the EU Union Civil Protection Mechanism and development of cross-border MCI plans such as the European burn mass casualty response plan might contribute to the increased cross-border focus in the European preparedness work [3, 29]. Norway as a member of the pan-European collaboration participates in both, although not a member of EU. In terms of possible future activation of NATO Article 5, NATO members must not only prepare for a military response, but also for the coordination, evacuation and treatment of injured soldiers [1].

The hospital geographically nearest to an MCI/MI event, is likely to receive a high influx of victims as seen during the Utøya incident and in international events [12, 30, 31]. Hence, MCI/MI competency among hospital staff at all trauma hospitals is a necessity. MCI/MI trainings is a crucial part of the hospitals preparedness system. The RHAs are mandated by law to train staff for disasters. The law does not specify the type of exercises, type of staff to be trained or the frequency [27, 28]. The MCI/MI exercise percentage within the last 2 years was lower in our study compared to data from major TC in Canada, Australia, England and New Zealand (65% vs 79%). Considerable variances were detected between the RHAs. Corresponding with our finding that MCI/MI exercise is an area of improvement, Jørgensen et al. detected that 54% of the study participants from Norwegian trauma hospitals had never participated in an MCI exercise [18]. Norway was hosting the NATO military exercise Trident Juncture in 2018. The civilian-military cooperation was tested during a national exercise involving prehospital and in-hospital services. The evaluation report indicated room for improvement regarding the Norwegian trauma system to meet NATO/Military requirements that will be placed on civilian hospitals in case of armed conflicts. Deficiencies were detected regarding war surgery competency, surgical personnel and equipment [32]. Focus on production, cost and hospital economy are likely to negatively impact the number of MCI/MI exercises. Full-scale exercises are costly and might influence the normal operation of the hospital. However, smaller table-top exercises can be easier to plan and conduct [33]. The documented benefits of MCI/MI exercises are undisputed [6, 7, 12, 34].

Less than half of the MCI/MI plan activations led to an evaluation process. Useful information about latent system errors, bottlenecks and shortages of competency and skills are possibly lost. Lack of evaluation routines after MCI/MI events was also detected among prehospital rescue services, indicating that missing out on potential learning points from MCI/MI events is not isolated to Norwegian hospitals [35]. Evaluations both after exercises and real events, can be a useful tools in the contingency work [34]. WHO recommends that a post-action report is provided to the hospital administration, emergency managers and appropriate stakeholders [36]. Many Norwegian trauma hospitals do not seem to adhere to the WHO advice.

Few Norwegian trauma hospitals had performed a Surge Capacity estimation within the last 5 years. Among major TC in Canada, Australia, England and New Zealand, variances were detected regarding maximum capacity estimation systems, indicating that Surge Capacity assessment is a global challenge [16]. Detailed determination of hospital Surge Capacity is of notable importance and can be assessed using the methodology described by Lennquist Montan [37]. Hirchberger et al. argue that the number of trauma teams is the key limiting factor [38]. Blimark et al. detected deficiencies in the surgical Surge Capacity among Swedish hospitals by assessing the number of surgical trauma teams, surgical theatres, ICU beds and intermediate care beds [19]. The authors were advocating for a national MCI/MI strategy in Sweden. In Norway there is no national benchmark regarding the number of surgical theatres or surgical trauma teams in relation to the population served. Based on our findings, it seems Norway also could benefit from a national MCI/MI strategy for hospital Surge Capacity. Future assessment of the Norwegian hospital Surge Capacity regarding available trauma teams, surgical theatres, ICU, intermediate beds and blood supply would be of interest for future study. Considering the current geopolitical situation, knowledge about hospital Surge Capacity on a pan-European level could be of interest.

Corresponding with the finding in our study, Söderin et al. identified that several primary in-hospital triage methods were used in MCI/MI situations in Sweden [17]. A national standardisation of in-hospital primary triage method based on anatomical and/or physiological criteria could be advocated for. This would ease communication around patient transferral between hospitals and standardise the primary triage across hospitals. As Bieler et al. emphasise, the management of gunshot wounds and blast injuries in an MCI/MI with unknown number of victims might demand a surgical focus based on tactical consideration and not only ATLS principles [38]. In the current European context this knowledge needs to be provided through MCI/MI education and trainings, as the exposure to these injuries in an MCI/MI setting is uncommon for most surgeons [4].

Despite that the RHAs are mandated by law to ensure PPE and provide important equipment and pharmaceuticals, supply management was detected as an area of improvement in the Norwegian in-hospital MCI/MI preparedness [28]. Our results are corresponding with the evaluation report after the Trident Juncture civilian-military NATO exercise, which detected shortages of equipment to manage war injuries [32]. The State of Health preparedness report from EU in 2022 highlights the importance of a “resilient chain of supplies” in terms of production and distribution, as weaknesses were detected during the Covid-19 pandemic [2]. The current geopolitical situation contributes to a sustained focus on supplies in the European disaster preparedness [2]. Disaster supply management is a concern also outside the European context [16].

As shown in this study and the corresponding Swedish study, a web-based survey could be used as a quality assurance tool to assess the MCI/MI preparedness on a national level [17]. By implementing minimal requirements and standards to benchmark towards, a modified survey could be used as a monitoring tool to follow up the contingency work.

## Limitation

Organisation of the contingency work is heterogenic and depending on the hospital size. This causes a risk of different interpretation of questions or that the questions did not feel adequate for the respondent’s hospital’s contingency organisation. Some respondents had several roles at the hospital and the role we asked each respondent to answer on behalf of was defined in the cover letter. The respondents were asked to answer on behalf of their organisation, and they were encouraged to seek information before answering. Inclusions of other functions than CRR was chosen to gain more profound knowledge of the hospitals contingency work as trauma team leaders and leaders of trauma departments might have more hands-on knowledge on the trauma and contingency work taking place. There is a risk that respondents are reluctant to release information about weaknesses in their hospitals contingency work. The study had good response rate and geographically coverage. This is probably due to close follow up of the respondents as well as the topic being highly relevant with gained public interest regarding climate changes and the current geopolitical situation. Some of the hospitals in Norway without trauma function, such as Elverum Hospital, might still receive trauma patients, although not defined as a NTC according to the NTP. The study lacks information of these hospitals. The questions in the survey could be adapted to fit other health systems. For the future it would be interesting to validate the method outside Scandinavia.

## Conclusion

A web-based survey could be a useful tool to gather information regarding in-hospital contingency work on a national level, both as a baseline and as a follow up tool. Many key elements are established in the Norwegian in-hospital contingency work, although several areas of improvement regarding MCI/MI education, training, exercises, triage, Surge Capacity and disaster storage were identified. Variances among the RHAs were detected, indicating a need for national minimum requirements and standards. Although, implementing minimal requirements and guidelines is probably insufficient if there is no follow up from regional and/or national authorities. Knowledge of Surge Capacity and disaster storage on a national level could be of interest from a pan-European perspective in today’s political situation.

## Supplementary Information

Below is the link to the electronic supplementary material.

Supplementary file1 (DOCX 42 KB)

## Data Availability

Due to security issues in regards to potential third party interest, data regarding hospitals contingency work can not be openly accessed. Data is stored in an secure server in Helse-Bergen.

## Abbreviations

ATLS: Advanced Trauma and Life support
CBRNE: Chemical, Biological, Radiological, Nuclear, Explosive
CRM: Crew Resource Management
CRR: Contingency responsible respondent/role or function as contingency coordinator
EU: European Union
HCG: Hospital Command Group
MCI: Mass Casualty Incident
MI: Major Incident
NATO: North Atlantic Treaty Organization
NTC: Non-Trauma Centre = Acute Hospital with trauma function
NTP: National Trauma Plan
RHA: Regional Health Authority
TC: Trauma Centre
WHO: World Health Organisation
