# Supplementary material for: A national study of in-hospital preparedness for Mass Casualty Incidents and disasters
Source: Eur J Trauma Emerg Surg. 2025 Jan 15;51(1):18. doi: 10.1007/s00068-024-02685-7 (PMC11735519; doi:10.1007/s00068-024-02685-7)
Supplement: Supplementary file 1 — Supplementary file1 (DOCX 42 KB) [file 68_2024_2685_MOESM1_ESM.docx]

**Appendix 1 Survey**

1. At which of the following trauma hospitals do you work?

⃝ Akershus University Hospital

⃝ Arendal Hospital

⃝ Bodø Hospital

⃝ Bærum Hospital

⃝ Drammen Hospital

⃝ Førde Hospital

⃝ Gjøvik Hospital

⃝ Hamar Hospital

⃝ Hammerfest Hospital

⃝ Haukeland University Hospital

⃝ Haugesund Hospital

⃝ Kalnes Hospital

⃝ Kirkenes Hospital

⃝ Kongsvinger Hospital

⃝ Kristiansand Hospital

⃝ Levanger Hospital

⃝ Lillehammer Hospital

⃝ Lofoten Hospital

⃝ Namsos Hospital

⃝ Mo I Rana Hospital

⃝ Molde Hospital

⃝ Odda Hospital

⃝ Ringerike Hospital

⃝ Sandnessjøen Hospital

⃝ St Olavs Hospital

⃝ Stavanger Hospital

⃝ Stord Hospital

⃝ Telemark Hospital

⃝ Ullevål Universty Hospital

⃝ UNN Harstad

⃝ UNN Narvik

⃝ UNN Tromsø

⃝ Tynset Hospital

⃝ Vestfold Hospital (Tønsberg)

⃝ Vesterålen Hospital

⃝ Volda Hospital

⃝ Voss Hospital

⃝ Ålesund Hospital

1. Which role at the hospital do you have?

⃝ Trauma coordinator/function as trauma coordinator

⃝ Leader of Trauma Department or Trauma Centre

⃝ Trauma Team Leader

⃝ Contingency coordinator or function as contingency responsible

⃝ Other (specify)

1. Does your hospital have a role/function responsible for the contingency work?

⃝ Yes, with dedicated time to perform the task.

⃝ Yes, but without dedicated time to perform the task.

⃝ No

⃝ Did not know.

1. If your hospital has a contingency coordinator, which occupational background has the beholder of the role?

⃝ Nurse

⃝ Medical Doctor

⃝ Other (specify)

1. If your hospital has a contingency coordinator, has the beholder MCI/MI disaster education, training or courses?

⃝ No formal education

⃝ Formal education (specify which education, where and when)

⃝ Course/training (specify what and when)

⃝ Not relevant as have no contingency coordinator.

⃝ Others (specify)

⃝ Did not know

1. If your hospital has a contingency coordinator, where in the hospital organisation chain is the function placed?
2. Does your hospital have a Medical Doctor responsible for disaster medicine preparedness?

⃝ Yes

⃝ No

⃝ Did not know.

1. If your hospital has a doctor responsible for disaster medicine preparedness, has the beholder of this function a MCI/MI disaster education, training or courses?

⃝ No formal education

⃝ Formal education (specify which education, where and when)

⃝ Course/training (specify what and when)

⃝ Not relevant as have no contingency coordinator.

⃝ Others (specify)

⃝ Did not know.

1. Does your hospital have a contingency committee?

⃝ Yes

⃝ No

⃝ Did not know.

1. Which functions are represented in the contingency committee at your hospital (multiple answer)?

⃝ Secretary/support

⃝ Administrator

⃝ Leader

⃝ Surgeon

⃝ Orthopaedic Surgeon

⃝ Security

⃝ Anaesthesia/ICU

⃝ Nurse

⃝ Psychological support

⃝ Communication and information

⃝ Others (specify)

⃝ Did not know.

1. Are there requirements in terms of MCI/MI education/training/courses for the members of the contingency committee?

⃝ No formal educational requirements

⃝ Formal education (specify which education, where and when)

⃝ Not relevant as have no contingency committee.

⃝ Others (specify)

⃝ Did not know.

1. Estimate the number of staff at your hospital that has specific training or courses in MCI/MI?

⃝ ≥10

⃝ 5-10

⃝ ≤ 5

⃝ None

⃝ Did not know.

1. Does your hospital have a trauma coordinator function/role?

⃝ Yes

⃝ No

⃝ Did not know.

1. Do your hospitals trauma team leaders fill the requirements in national trauma plan regarding minimum 4 years of surgery, trauma team training, ATLS and live tissue courses in damage control surgery?

⃝ Yes, all Trauma Team Leaders

⃝ Yes, most Trauma Team Leaders

⃝ Few of the Trauma Team Leaders fulfil the requirements

⃝ None

⃝ Did not know.

1. Do your hospital conduct certification of trauma team leaders?

⃝ Yes (specify how the certification is conducted)

⃝ No

⃝ Did not know.

1. In an MCI/MI event how many members do your trauma team consist of?

⃝ 2

⃝ 3

⃝ 4

⃝ 5

⃝ > 6

⃝ Did not know.

1. Which professions are represented in the modified MCI/MI trauma team?

⃝ Surgeon/orthopaedic surgeon

⃝ Anaesthetic doctor

⃝ Anaesthetic nurse

⃝ Emergency Department nurse

⃝ Operating nurse

⃝ Others (specify)

⃝ Did not know.

1. Which communication tools are used in the trauma team during single or MCI/MI situation (multiple answers)?

⃝ MIST

⃝ ISBAR

⃝ ATLS

⃝ Closed loop

⃝ Time-out

⃝ Methane

⃝ Others (specify)

⃝ Did not know.

1. How is the documentation done during a trauma team reception at your hospital?

⃝ Trauma paper file

⃝ Trauma paper file + immediate digital documentation in patient file

⃝ Trauma paper file + Anaesthetic paper file

⃝ Digital documentation + Anaesthetic paper file

⃝ Trauma paper file + Anaesthetic paper file + digital documentation in patient file in “cold phase”

⃝ Others (specify)

⃝ Did not know.

1. How extensive is the overall contingency plan at your hospital?

⃝ The hospital has one overall contingency plan.

⃝ Several Departments have their own contingency plans that are synchronised and coordinated.

⃝ Several Departments have their own contingency plans that are NOT synchronised and coordinated.

⃝ Did not know.

1. How many pages is the overall contingency plan at your hospital?

⃝ 1

⃝ 2

⃝ 3

⃝ 4

⃝ 5-10

⃝ 11-20

⃝ 21-30

⃝ 31-40

⃝ ≥ 41

⃝ Did not know.

1. Describe shortly how the contingency plan for MCI/MI is organised?
2. When was the overall contingency plan last revised/updated?

⃝ < 1 year

⃝ 1-2 years

⃝ 2-3 years

⃝ > 3 years

⃝ Did not know.

1. When was the contingency plan for MCI last revised/updated?

⃝ < 1 year

⃝ 1-2 years

⃝ 2-3 years

⃝ > 3 years

⃝ Did not know.

1. Is the contingency plan for MCI coordinated with neighbouring hospitals contingency plan?

⃝ Yes (specify which hospitals)

⃝ No

⃝ Did not know.

1. Who reads the overall contingency plan?

⃝ All newly employees should read the contingency plan.

⃝ All hospital staff are required to read the contingency plan in accordance with local guideline.

⃝ The hospital has no requirements regarding which staff should read the contingency plan.

⃝ Is up to each individual staff to decide if he/she should read the contingency plan

⃝ Others (specify)

⃝ Did not know.

1. Which scenario are described in the overall contingency plan?

⃝ Fire

⃝ MCI/MI

⃝ Natural disaster

⃝ CBRNE

⃝ Epidemic

⃝ Hypothermia

⃝ Inhalation injury

⃝ Evacuation of the hospital

⃝ Computer network error /Mobile phone breakdown

⃝ Psychological trauma

⃝ Security threat towards the hospital

⃝ No scenarios described

⃝ Others (specify)

⃝ Did not know.

1. Does the overall contingency plan describe which scenarios it is dimensioned for?

⃝ Yes (specify)

⃝ No

⃝ Did not know.

1. Which of the following roles/functions has an action card in case of MCI/MI (multiple answers)?

⃝ Emergency room doctor

⃝ Triage doctor

⃝ Surgoen ED

⃝ Surgeon OR

⃝ Anaesthetic doctor ED

⃝ Anaesthetic doctor ICU

⃝ Anaesthetic doctor Postoperative care

⃝ Anaesthetic doctor OR

⃝ Nurse ED

⃝ Nurse OR

⃝ Nurse ICU

⃝ Nurse postoperative

⃝ Nurse ward

⃝ Radiologist

⃝ Radiograph

⃝ Doctor ward

⃝ Bioengineer

⃝ Blood bank

⃝ Security

⃝ Medical technical support

⃝ Porter

⃝ Psychological support personnel

⃝ Technical support

⃝ Secretary support service

⃝ Hospital Command Group

⃝ The hospital does not operate with action cards.

⃝ Others (specify)

⃝ Did not know.

1. How often does your hospital run exercises to practise the contingency plan?

⃝ More often than yearly

⃝ Yearly

⃝ Every second year

⃝ Longer interval than every second years

⃝ The hospital runs no exercises.

⃝ Did not know.

1. Which staff participate in the contingency exercises at your hospital?

⃝ All staff involved in the trauma team.

⃝ We train staff on rotation in yearly exercises.

⃝ Normally it is staff with special interest in contingency that participate

⃝ Others (specify)

⃝ The hospital runs no exercises.

⃝ Did not know.

1. Does your hospital train different disaster scenarios?

⃝ Yes, the hospital train trauma and other scenarios (gas leakage, fire etc)

⃝ Yes, but only physical trauma

⃝ No, the hospital does not train different sceanrios

⃝ Others (specify)

⃝ The hospital runs no exercises.

⃝ Did not know.

1. How does your hospital evaluate disaster exercises?

⃝ Structured evaluation within the contingency committee

⃝ Questionnaire after the exercise to test the knowledge of staff.

⃝ No evaluation takes place.

⃝ Others (specify)

⃝ The hospital runs no exercises.

⃝ Did not know.

1. If your hospital evaluated disaster exercises, how is the results from the evaluation used?

⃝ The evaluation is used to improve the contingency plan.

⃝ The evaluation is used to assess the exercise.

⃝ The evaluation is used to assess the staff competency for MCI/MI

⃝ The hospital does not conduct evaluation.

⃝ Others (specify)

⃝ The hospital runs no exercises.

⃝ Did not know.

1. How is the Hospital Command Group organised during a severe MCI/MI event at your hospital?

⃝ The Hospital Command Group is multidisciplinary group and has received contingency training.

⃝ The Hospital Command Group is multidisciplinary group, but they have not received contingency training.

⃝ The hospital does not have a Hospital Command Group

⃝ Others (specify)

⃝ Did not know.

1. Which function is represented in the Hospital Command Group at your hospital?

⃝ Secretary/support

⃝ Administrator

⃝ Leader

⃝ Human Resources

⃝ Finance

⃝ Surgeon/Orthopaedic Surgeon

⃝ Security

⃝ Anaesthesia/ICU

⃝ Nurse

⃝ Supply

⃝ Communication and information

⃝ Others (specify)

⃝ Did not know.

1. Does the Hospital Command Group work after a specified method (NATO or other)?

⃝ Yes (specify)

⃝ No

⃝ Did not know.

1. Does the Hospital Command Group participate in (internal and/or external) disaster exercises?

⃝ Yes, internal exercises for the Hospital Command Group

⃝ Yes, exercises involving other departments at the hospital.

⃝ Yes, internal exercises for the Hospital Command Group and exercises involving other departments at the hospital.

⃝ No

⃝ Did not know.

1. When was the last time the Hospital Command Group participated in an MCI exercise at your hospital?

⃝ < 1 year

⃝ 1-2 years

⃝ 2-3 years

⃝ > 3 years

⃝ Did not know.

1. Is the Hospital Command Group obliged to have training in staff methodology?

⃝ Yes, for all members

⃝ Yes, for some members

⃝ No

⃝ Did not know.

1. Which communication devices is the hospital using in an MCI?

⃝ DECT/calling

⃝ Mobile phones

⃝ National emergency radio network

⃝ No back up system

⃝ Others (specify)

⃝ Did not know.

1. Does the hospital have a system in place to update the contact information (mobile number) of staff that is planned alarmed in an MCI/MI?

⃝ Yes, a person/role is responsible for updating the list

⃝ The hospital updates the lists, but no system for this is established.

⃝ Others (specify)

⃝ Did not know.

1. How is relevant staff alarmed during office hours/on duty?

⃝ SMS (specify)

⃝ Phone call to private mobile phone (specify)

⃝ Phone call to job phone (specify)

⃝ Loudspeaker

⃝ A dedicated person calls from each department

⃝ Did not know.

1. How is relevant staff alarmed out of office hours/off duty?

⃝ Phone call to private mobile phone (specify)

⃝ A dedicated person calls from each department.

⃝ Others (specify)

⃝ Did not know.

1. Where (physical location) in the hospital is primary triage conducted?

⃝ Ambulance entrance

⃝ Emergency Department

⃝ Other defined place inside the hospital (specify)

⃝ Triage place is not defined.

⃝ Did not know.

1. If conducted, where is secondary triage conducted in the hospital?

⃝ Emergency Department

⃝ Other defined place inside the hospital (specify)

⃝ The Trauma Team decide.

⃝ Did not know.

1. Which systems is used for primary triage in MCI/MI?
2. If performed, which system is used for secondary triage?
3. Does your hospital have an estimation of maximum capacity/surge-capacity for MCI/MI?

⃝ Yes

⃝ No

⃝ Did not know.

1. Describe in a few words how the estimation of capacity (surge-capacity) was conducted at your hospital?
2. When was the last maximum capacity/surge-capacity assessment conducted at your hospital?

⃝ Yes (specify the year)

⃝ No maximum capacity/surge-capacity assessment conducted.

⃝ Did not know.

1. Which of the following is included in your hospital is included in the maximum capacity/surge-capacity assessment?

⃝ ED

⃝ ICU

⃝ OR

⃝ Postoperative

⃝ Ward

⃝ Radiology

⃝ Supply

⃝ Disinfection central

⃝ Human Resources

⃝ Others (specify)

⃝ No maximum capacity/surge-capacity assessment conducted.

⃝ Did not know.

1. Does your hospital have a plan for referral of patients in an MCI/MI?

⃝ Yes

⃝ No

⃝ Did not know.

1. Has your hospital been involved in mass inflow of patients/disaster exercises involving transfer of patients to other hospitals?

⃝ Yes

⃝ No

⃝ Did not know.

1. Does your hospital have a disaster storage?

⃝ Yes

⃝ No

⃝ Did not know.

1. How frequent is the disaster storage revised?

⃝ Yearly

⃝ Every second year

⃝ Less frequent than every second year

⃝ The hospital has no disaster storage.

⃝ Did not know.

1. Which criteria are used to decide the contents of the disaster storage?

⃝ The contents are based on the dimension of the different scenarios as described in the contingency plan.

⃝ The disaster storage content is not defined.

⃝ The hospital has no disaster storage.

⃝ Did not know.

1. Does your hospital have a system to increase the in-patient capacity?

⃝ Yes (specify)

⃝ No

⃝ Did not know.

1. Where in the hospital are the less severely injured patients placed after admission in a situation with mass inflow of patients?

⃝ In several wards

⃝ In one ward if possible (specify which ward)

⃝ Others (specify)

⃝ Did not know.

1. When was the last time your hospital activated the contingency plan for MCI/MI?

⃝ Within last 2 years

⃝ 2-5 years

⃝ 5-10 years

⃝ Never

⃝ Did not know.

1. If the hospital activated the MCI/MI plan, was an evaluation performed to assess the hospitals performance?

⃝ Yes (specify how)

⃝ No

⃝ Did not know.

1. If the hospital activated the contingency plan, did the activation result in changes in the contingency plan?

⃝ Yes, changes in the contingency plan were made based on the deficiencies detected

⃝ No, the contingency plan worked well

⃝ No, consideration was made in terms of changing the contingency plan based on experiences from the event

⃝ Did not know.

1. Comments?
